# MGDG extracted from spinach enhances the cytotoxicity of radiation in pancreatic cancer cells

**DOI:** 10.1186/s13014-016-0729-0

**Published:** 2016-11-22

**Authors:** Hiroaki Akasaka, Yoshiyuki Mizushina, Kenji Yoshida, Yasuo Ejima, Naritoshi Mukumoto, Tianyuan Wang, Sachiko Inubushi, Masao Nakayama, Yuki Wakahara, Ryohei Sasaki

**Affiliations:** 1 Division of Radiation Oncology, Kobe University Graduate School of Medicine, Chuo-ku, Kobe, Hyogo 650-0017 Japan; 2 Graduate School of Agriculture, Shinshu University, Minamiminowa-mura, Kamiina-gun, Nagano, 399-4598 Japan

**Keywords:** Monogalactosyl diacylglycerol (MGDG), Radiation, Pancreatic cancer, Radiosensitizer, Apoptosis

## Abstract

**Background:**

In our previous study, monogalactosyl diacylglycerol (MGDG) purified from spinach was found to have cytotoxic effects in human cancer cell lines. This study further assessed whether MGDG can enhance the cytotoxic effects of radiation in human pancreatic cancer cells in vitro and in vivo.

**Methods:**

Glycoglycerolipids from spinach including MGDG were extracted from dried spinach. The cytotoxicity of MGDG were evaluated by the MTT assay using four human pancreatic cancer cell lines (MIAPaCa-2, AsPC-1, BxPC-3 and PANC-1) and normal human dermal fibroblasts (NHDFs). The effects of radiation and MGDG alone or in combination in MIAPaCa-2 cells was analyzed with the colony forming and apoptosis assays, western blotting and cell cycle and DNA damage analyses (γ-H2AX foci staining and comet assay). The inhibitory effects on tumor growth were assessed in a mouse xenograft tumor model.

**Results:**

MGDG showed dose- and time-dependent cytotoxicity, with half-maximal inhibitory concentrations (IC_50_) in PANC-1, BxPC-3, MIAPaCa-2 and AsPC-1 cells at 72 h of 25.6 ± 2.5, 26.9 ± 1.3, 18.5 ± 1.7, and 22.7 ± 1.9 μM, respectively. The colony forming assay revealed fewer MIAPaCa-2, BxPC-3 and AsPC-1 cell colonies upon treatment with both MGDG and radiation as compared to irradiation alone (*P* < 0.05). The combination of MGDG and radiation induced a higher proportion of apoptosis in MIAPaCa-2 cells; this effect was associated with increased mitochondrial release of cytochrome c and activation of cleaved poly (ADP-ribose) polymerase and caspase-3. DNA damage was detected and DNA repair mechanisms were more frequently impaired in cells receiving the combination treatment as compared to either one alone. Tumor growth was inhibited to a greater degree in mice treated by intratumoral injection of MGDG combined with irradiation as compared to either one alone (*P* < 0.05).

**Conclusions:**

This is the first report demonstrating that MGDG enhances the cytotoxicity of radiation to induce apoptosis of cancer cells in vitro and in vivo. Our findings indicate that this therapeutic combination can be an effective strategy for the treatment of pancreatic cancer.

## Background

Pancreatic cancer remains a major public health issue as the third leading cause of cancer-related death in Europe, the fourth in the United States, and the fifth (men) or sixth (women) in Japan [1–3]. Recent in vitro and in vivo studies have shown that consumption of vegetables and fruits with chemopreventive components can reduce cancer risk [4–6]. The chloroplast thylakoid membrane of higher plants contains glycoglycerolipids such as monogalactosyl diacylglycerol (MGDG), digalactosyl diacylglycerol (DGDG) and sulfoquinovosyl diacylglycerol (SQDG) [7]; these compounds have potential anti-cancer functions including inhibition of DNA polymerase and suppression of cancer cell proliferation [8], with MGDG showing more potent anti-tumorigenic and anti-inflammatory activity than the others [9]. Spinach is a major source of glycoglycerolipids and has the highest MGDG content among vegetables, fruits and grains tested to date [10–12].

Various approaches have been used to improve the survival rate of pancreatic cancer patients, including adjuvant chemotherapy [13–15], preoperative chemoradiotherapy [16–18], and induction chemotherapy followed by chemoradiotherapy [19, 20]. Our previous study showed that MGDG enhanced the cytotoxic effects of gemcitabine (GEM)—a key drug for treating pancreatic cancer—possibly by selectively inhibiting mammalian replicative DNA polymerases, specifically pol γ [21].

The present study further investigated whether MGDG might enhance the effects of radiation on human pancreatic cancer cell lines in vitro and in vivo.

## Methods

### Isolation of MGDG from spinach

The spinach (*Spinacia oleracea*) subspecies Anna was used in this study. Dried spinach was extracted with ethanol; the extract was diluted in 70% aqueous ethanol and subjected to Diaion HP-20 column chromatography (Sigma-Aldrich, St. Louis, MO, USA), then eluted with 95% aqueous ethanol. The eluate was evaporated to dryness; the residue was redissolved in chloroform and the resultant solution was subjected to PSQ60B silica gel column chromatography (Fuji Silysia Chemical, Tokyo, Japan). After washing with chloroform/ethyl acetate (1:1 v/v), the column was eluted with ethyl acetate and the eluate was purified using a Sep-Pak C_18_ cartridge (Waters, Milford, MA, USA) that was then eluted with methanol. The MGDG fraction was evaporated, yielding pure MGDG (~98%). The purity was confirmed by normal-phase silica gel high-performance liquid chromatography (Shiseido, Tokyo, Japan) coupled to an evaporative light scattering detector (M&S Instruments, Osaka, Japan), with chloroform/methanol (1/1, v/v) used as the eluent.

### Cell culture and viability assessment

MIAPaCa-2, PANC-1, BxPC-3 and AsPC-1 human pancreatic cancer cell lines as well as Raji and HL60 cells were obtained from the American Type Culture Collection (Manassas, VA, USA) and cultured in Roswell Park Memorial Institute 1640 medium supplemented with 10% fetal bovine serum, penicillin (100 U/ml), streptomycin (100 μg/ml). HCT116 colon carcinoma cell lines with wild-type p53 (HCT116 p53^+/+^) and their isogenic derivatives lacking p53 (HCT116 p53^−/−^) were a gift from Dr. Bert Vogelstein (Johns Hopkins University, Baltimore, MD, USA). The cells were maintained in McCoy’s 5A medium. Primary normal human dermal fibroblasts (NHDFs) were purchased from Cell Systems Corp. (Kirkland, WA, USA) and maintained according to the supplier’s instructions. The cytotoxicity of MGDG was evaluated with the 3-(4, 5-dimethylthiazol-2-yl)-2, 5-diphenyltetrazolium bromide (MTT) assay. The cytotoxicity when combined with irradiation was assessed with the colony forming assay. For the MTT assay, cells were treated with 0, 2, 5, 10, 20, 50 or 100 μM MGDG or corresponding doses of dimethyl sulfoxide (DMSO) for 24, 48 or 72 h at 37 °C in a humidified atmosphere of 5% CO_2_; 2 h after adding MTT solution (0.6 mg/ml in Milli-Q-purified water), cells were lysed in 200 μl of fresh DMSO. For the colony forming assay, MIAPaCa-2, BxPC-3 and AsPC-1 cells were treated with 40–60 μM MGDG or 0.8% DMSO for 24 h, and then exposed to 0, 2, 4 and 8 Gy of radiation. After 9–12 days, colonies were fixed with a solution of 10% methanol and 20% acetic acid, stained with Methylene Blue and counted under a light microscope.

### X-ray irradiation

X-ray irradiation was performed using a MBR-1505R2 instrument (Hitachi, Tokyo, Japan) at a voltage of 150 kV and a current of 5 mA with a 1-mm-thick aluminum filter (0.5 Gy/min at the target) for in vitro and in vivo studies. Mice were anesthetized by intraperitoneal administration of somnopentyl (0.1 mg/g body weight) and were then anesthetized and immobilized in a customized harness that exposed the implanted tumors while shielding the remainder of the body with lead during irradiation.

### Analysis of apoptotic cells

Apoptosis was evaluated based on DNA fragmentation using the APO-Direct Assay Staining kit (BD Biosciences, San Diego, CA, USA) as previously described [22]. In this assay, DNA breaks are labeled with fluorescein isothiocyanate (FITC)-2′-deoxyuridine-5′-triphosphate and cells are analyzed by flow cytometry. MIAPaCa-2 cells were treated with MGDG (25 μM) alone for 24 h, radiation (5 Gy) alone for 12 h, or with a combination of both. The cells were harvested by trypsinization, washed with phosphate-buffered saline (PBS), and then fixed in 1% paraformaldehyde for 15min followed by 70% ethanol overnight at −20 °C. A DNA labeling solution containing FITC was added for 30min; the cells were the resuspended in PBS, and apoptosis was detected by flow cytometry on a FACS Calibur instrument (Becton Dickinson, Franklin Lakes, NJ, USA). Data were analyzed with CellQuest software (Becton Dickinson), and apoptotic cells were quantified as a percentage of the total number of cells.

### Western blot analysis

MIAPaCa-2 cells were seeded at a density of 2 × 10^6^/well in a 6-well plate for 24 h, then washed with PBS and incubated with various concentrations of MGDG for 24 h. Cells were washed twice with PBS and lysed in lysis buffer composed of 20 mM HEPES (pH 7.5), 10% glycerol, 1.5 mM MgCl_2_, 1 mM TritonX-100, and protease inhibitor cocktail (Roche Life Science, Tokyo, Japan) followed by boiling for 5min. To isolate cytosolic and mitochondrial fractions, MIAPaCa-2 cells were harvested by scraping on ice, and resuspended in 500 μl buffer A composed of 20 mM HEPES (pH 7.5), 10 mM KCl, 1.5 mM MgCl_2_, 1 mM EDTA, 1 mM EGTA, and protease inhibitor cocktail. After incubation on ice for 1 h, cells were lysed by repeated passage (20–30 times) through a 27-gauge needle. The lysates were centrifuged at 750 × *g* for 5min at 4 °C and the supernatant was centrifuged at 10,000 × *g* for 15min at 4 °C. The mitochondrial pellet was washed once in buffer A and lysed in Laemmli sample buffer. The supernatant was centrifuged at 100,000 × *g* for 30min at 4 °C to obtain the cytosolic fraction. Protein concentrations were measured with the bicinchoninic acid protein assay kit (Pierce Biotechnology, Rockford, IL, USA) according to the manufacturer’s protocol. Proteins were separated by sodium dodecyl sulfate-polyacrylamide gel electrophoresis and then transferred to nitrocellulose membranes that were blocked with 5% non-fat milk in PBS and probed overnight at 4 °C with primary antibodies against the following proteins: actin (Santa Cruz Biotechnology, Dallas, TX, USA), poly (ADP-ribose) polymerase (PARP) (Cell Signaling Technology, Danvers, MA, USA), caspase-3 (Cell Signaling Technology), pro-caspase-3 (GeneTex, Irvine, CA, USA), B cell lymphoma (Bcl)-2 (Santa Cruz Biotechnology), and Bcl-2-associated X protein (Bax) (Santa Cruz Biotechnology). Immunoreactivity was detected with an enhanced chemiluminescence kit (GE Healthcare, Little Chalfont, UK) and protein bands were visualized using an Amersham Imager 600 (GE Healthcare). Signal intensity was quantified using Multi Gauge v.3.0 software (Fujifilm, Tokyo, Japan).

### Cell cycle analysis

The effect of MGDG on the cell cycle was evaluated by flow cytometry as previously described [23]. Briefly, MIAPaCa-2 cells (3 × 10^5^ cells in a 25-ml flask) were treated with 40 μM MGDG, 8 Gy of radiation, or a combination of both for 24 h. Cells were irradiated within 12 h of adding MGDG and incubated for 12 h, then fixed on ice for 30min in PBS (pH 7.4) containing 2% formaldehyde and stored at −20 °C until analysis. Cells were washed and incubated for 15min in phosphate citric acid buffer composed of 20% Triton X and 5 mg/ml ribonuclease A in PBS, then resuspended in 50 mg/ml propidium iodide for at least 15min at room temperature in the dark; the DNA content of the samples was analyzed by flow cytometry using a FACScan instrument (Becton Dickinson) with a 488-nm laser run at 15 mW and a 585/420-nm bandpass filter. At least 20,000 events were acquired using CellQuest software (Becton Dickinson). The experiment was performed at least twice. The G1, S and G2 fractions were identified by selecting the areas consisting of living cells and excluding those containing dead cells.

### Detection of DNA damage in vitro

Induction of DNA damage was investigated by detecting phosphorylated histone 2AX (γ-H2AX)-positive foci by immunocytochemistry [24]. Briefly, MIAPaCa-2 cells were subcultured in 35-mm dishes and treated with 40 μM MGDG for 1 h and/or 8 Gy of radiation. Cells were then fixed in 4% paraformaldehyde in PBS for 20min, permeabilized with 0.1% Triton X-100 in PBS for 5min, and blocked in 5% bovine serum albumin in PBS for 60min. The cells were incubated with rabbit anti γ-H2AX antibody (1:200; Cell Signaling Technology, Danvers, MA, US) overnight at 4 °C, followed by incubation with tetramethyl rhodamine isothiocyanate-conjugated anti-rabbit secondary antibody (1:20; Dako, Glostrup, Denmark) for 90min at room temperature. Nuclei were stained with 4′,6-diaidino-2- phenylindole, and cells were visualized with a fluorescence microscope (BZ-9000; Keyence, Osaka, Japan). Nuclear γ-H2AX foci in 200 cells in each treatment group were manually counted, and data are presented as the mean ± standard deviation of three random fields.

### Comet assay for detection of DNA repair impairment

The alkaline comet assay was performed using a kit (Trevigen, Gaithersburg, MD, USA) according to the manufacturer’s instructions. Briefly, 250 ml of 0.65% normal agarose was prepared and a drop was placed on a frosted slide, covered with a coverslip and allowed to solidify. The cell suspension (100 ml) was mixed at 1:10 with 0.5% low-melting-point agarose and a 100 μl volume of the mixture was pipetted onto the slides and allowed to solidify. A final layer of 0.5% low-melting-point agarose was added, and slides were then immersed for 1 h at 4 °C in the dark in cold lysis solution composed of 2.5 M NaCl, 100 mM EDTA, 300 mM NaOH, 10 mM Tris, and 34 mM N-lauroylsarcosine (pH 10), with 10% DMSO and 1% Triton X-100 added just before use. Slides were placed in a submarine-type electrophoresis tank containing 300 mM NaOH and 1 mM EDTA (pH 13.5) for 15min. Electrophoresis was then carried out at 0.8 V/cm for 15min. Slides were rinsed three times with neutralization buffer (0.5 M Tris, pH 7.4) and stained with a solution of 1 μl SYBR Green Gold in 30 ml Tris/EDTA buffer.

### Evaluation of in vivo tumor growth-inhibitory effect

Male BALB/cAJcl-nu/nu mice (4 weeks old; *n* = 10 per group) were used for the xenograft model. Experiments were carried out in strict accordance with institutional ethics guidelines. Each mouse received a subcutaneous intratumoral injection of 2 × 10^7^ MIAPaCa-2 cells resuspended in the Matrigel (BD Biosciences) and were then randomly divided into the following four groups: control, MGDG (injection with 2 mg MGDG solution twice on alternate days to ensure good penetration into the tumors), irradiation (5 Gy), and the combination of both. For this procedure, MGDG was used at a concentration of 50 mg/ml (62.5 mM) in solvent, and 4 mg MGDG solution (two injections of 2-mg MGDG solution) were used to prepare 50 μM MGDG. To assess the tumor growth-inhibitory effect of MGDG and/or radiation, tumor size was measured two or three times a week by calculating the volume using the formula L × W^2^ × (π/6), where L and W are the longest and shortest diameters of the tumor, respectively. All animal experiments were approved by the Institutional Animal Care and Use Committee (permission no. 100605R1) and were performed according to Kobe University Animal Experimentation Regulations.

### Statistical analysis

Data are presented as mean ± standard error. Differences between groups were evaluated with the Student’s *t* test. Data were considered statistically significant at *P* < 0.05.

## Results

### Cell viability

**Fig. 1:**
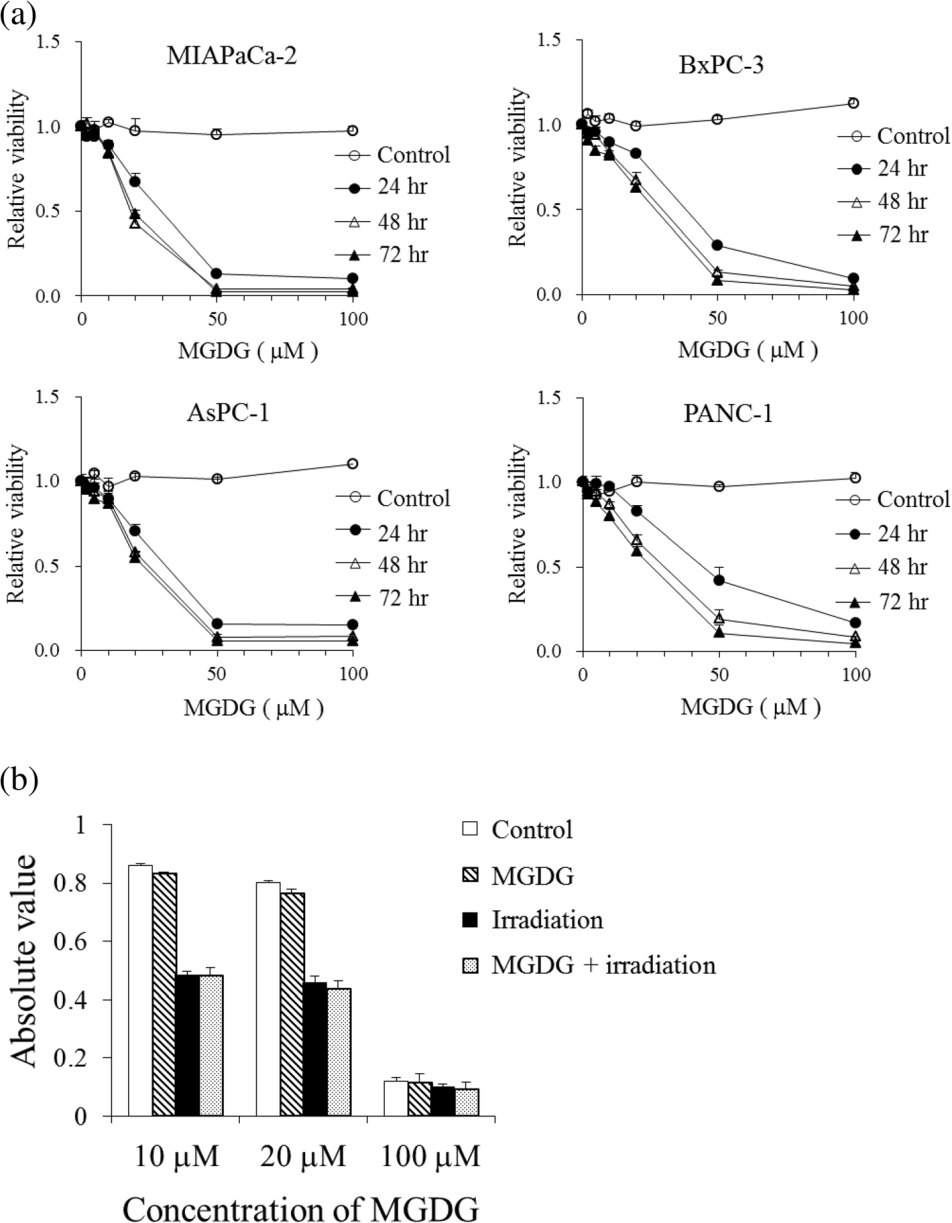
**a** Cytotoxic effects of MGDG on four human pancreatic cancer cells, as determined by the MTT assay. **b** Effects of MGDG, radiation and their combination on NHDFs, as evaluated by the MTT assay

**Fig. 2:**
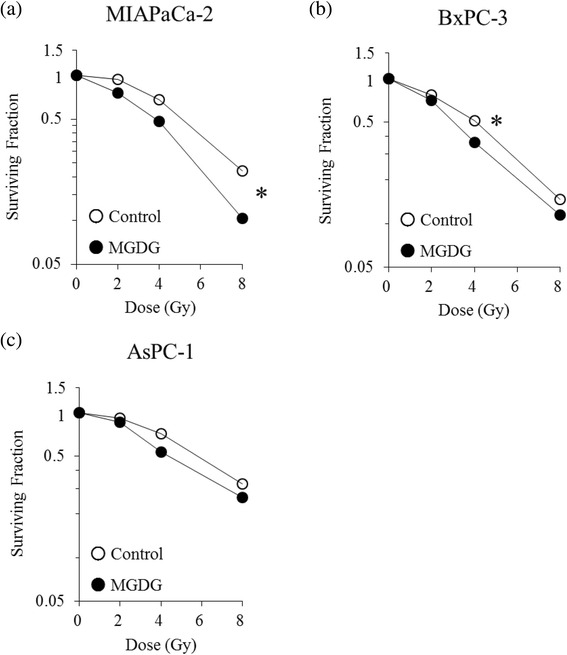
Colony-forming assay of MIAPaCa-2, BxPC-3 and AsPC-1 cells after exposure to graded doses of X-ray radiation combined with MGDG. *P < 0.05

Compared to DGDG or SQDG, MGDG showed distinct cytotoxic effects on human cancer cell growth (Table 1). MGDG showed dose- and time-dependent cytotoxicity in all four human pancreatic cancer cell lines, as determined with the MTT assay (Fig. 1a). The half-maximal inhibitory concentrations (IC_50_) in MIAPaCa-2, BxPC-3, AsPC-1 and PANC-1 cells at 72 h were 18.5 ± 1.7, 26.9 ± 1.3, 22.7 ± 1.9, and 25.6 ± 2.5 μM, respectively. In contrast, MGDG showed almost no cytotoxicity in NHDFs, and combined MGDG treatment and irradiation did not have a synergistic effect in NHDFs as compared to irradiation alone (Fig. 1b). The colony forming assay revealed fewer MIAPaCa-2, BxPC-3 and AsPC-1 cell colonies upon treatment with the combination treatment as compared to irradiation alone (*P* < 0.05) (Fig. 2).

**Table 1:** Cytotoxic effects of glycoglycerolipids from the spinach on human cancer cell growth

		IC_50_ (μM)		
Cell type	Tissue origin	DGDG	SQDG	MGDG
HCT116 p53^+/+^	Colon	22	78	13
HCT116 p53^−/−^	Colon	30	180	16
HL60	Leukemia	27	144	5
Raji	Lymphoma	22	114	5
PANC-1	Pancreas	103	48	11
MIAPaCa-2	Pancreas	16	66	4
Median		26	96	8

*DGDG* digalactosyl diacylglycerol, *IC*_*50*_ half-maximal (50%) inhibitory concentration, *MGDG* monogalactosyl diacylglycerol, *SQDG* sulfoquinovosyl diacylglycerol

### Induction of apoptosis

**Fig. 3:**
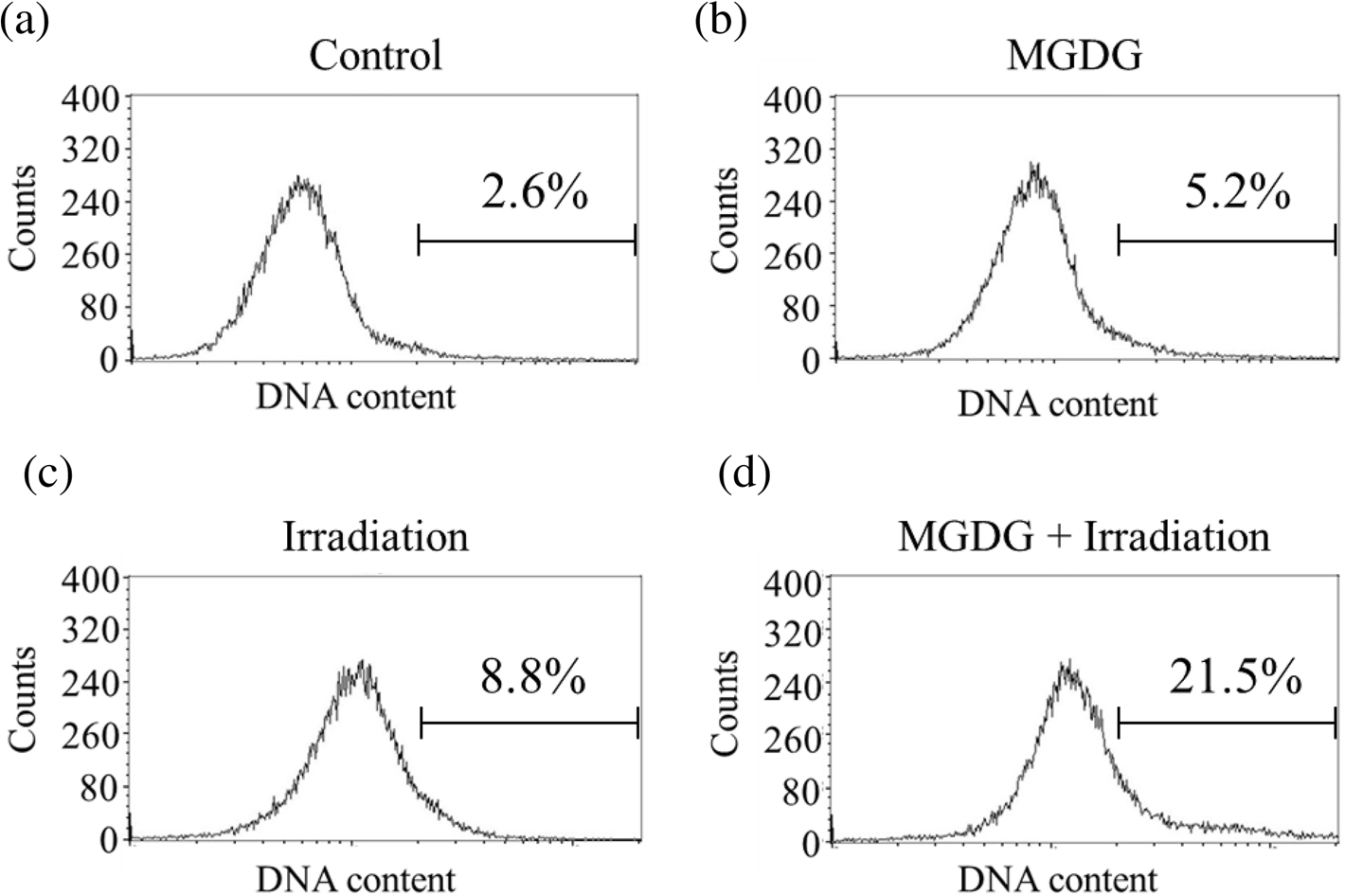
Induction of apoptosis after MGDG, irradiation and their combination in MIAPaCa-2 cells. **a** Control, **b** 25 μM MGDG, **c** 5 Gy radiation, **d** combination of 25 μM MGDG and 5 Gy radiation

The single treatments increased the rate of apoptosis (5.2% for MGDG and 8.8% for radiation), while the combination of both yielded a higher proportion of apoptotic cells (21.5%), suggesting that they had a synergistic effect on apoptosis induction (Fig. 3).

**Fig. 4:**
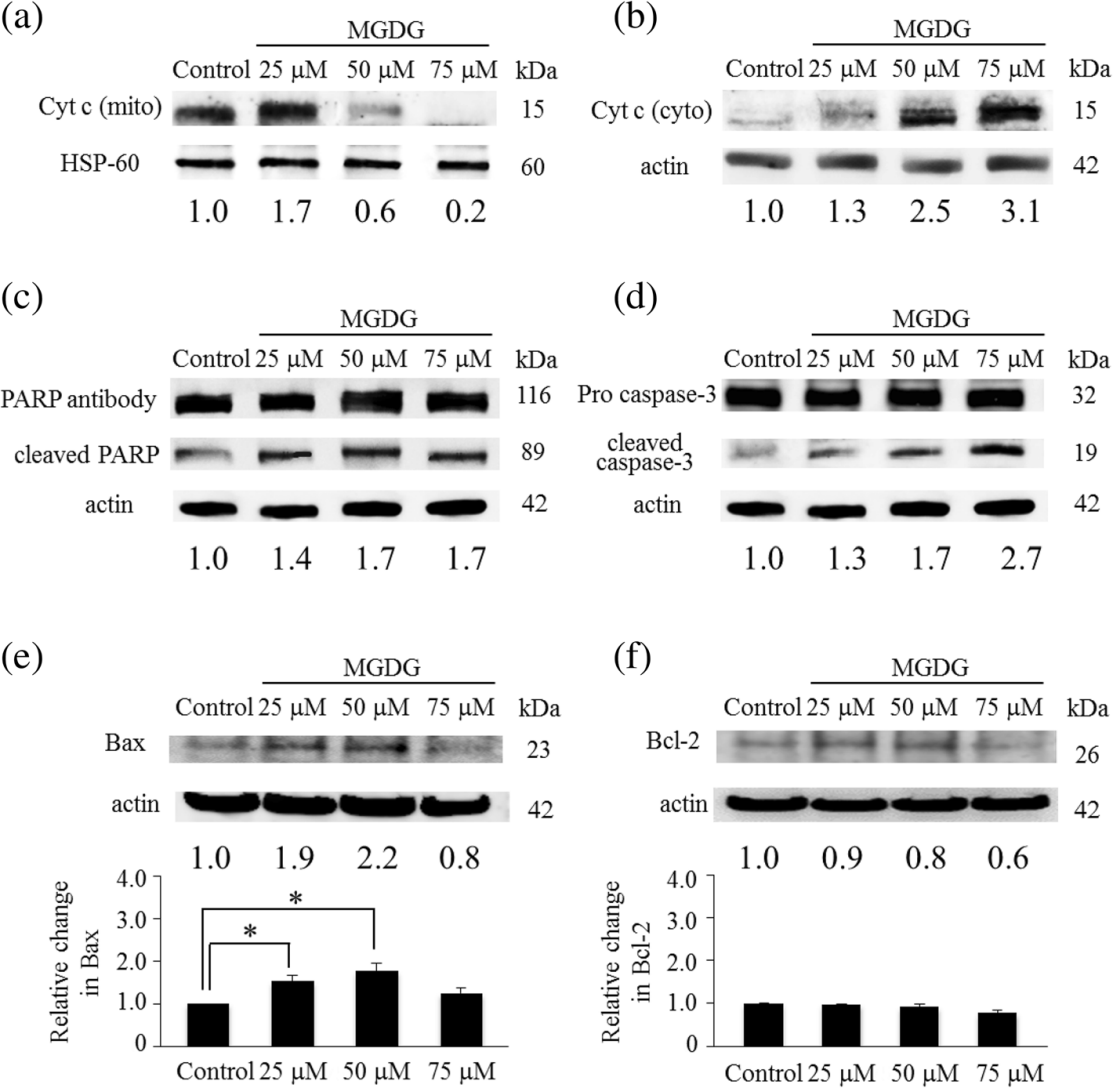
Molecular events underlying apoptosis in MIAPaCa-2 cells treated with MGDG. **a** Cytochrome c in mitochondria, **b** Cytochrome c in the cytosol, **c** PARP and cleaved PARP, **c** Pro caspase-3 and cleaved caspase-3, **e** Bax and **f** Bcl-2 levels were determined by western blotting. Protein levels were evaluated relative to HSP-60 or actin. *P < 0.05

Molecular events underlying apoptosis in MIAPaCa-2 cells treated with MGDG were investigated by the western blotting. Cytochrome c release from mitochondria to the cytosol increased in a concentration-dependent manner in the presence of 50 and 75 μM of MGDG; that is, cytochrome c level was reduced in mitochondria and increased in the cytosol relative to control cells (Fig. 4a, b). The activation of cleaved PARP and cleaved caspase-3 was also increased by MGDG treatment (Fig. 4c, d), with the latter possibly resulting from increased mitochondrial release of cytochrome c. Bax was also upregulated in a dose-dependent manner at 25 an 50 μM, whereas Bcl-2 level remained largely unaffected (*P* < 0.05) (Fig. 4e, f). A possible reason for slight decrease in Bax and Bcl-2 levels at 75 μM MGDG is cell loss caused by severe MGDG toxicity. Results from the western blot analysis were compared with the Student’s *t* test [22].

### DNA damage and DNA repair impairment

**Fig. 5:**
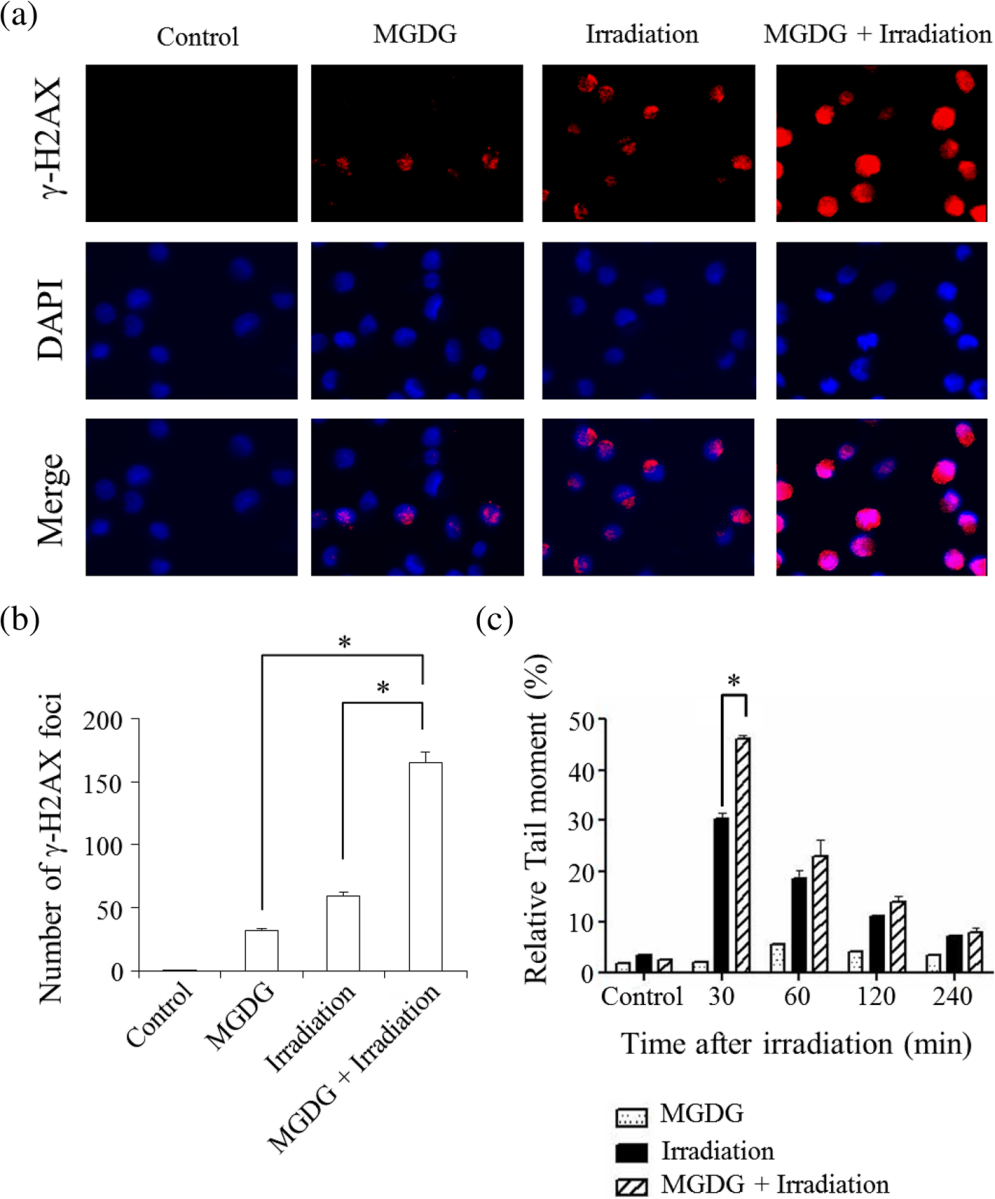
DNA damage and repair in MIAPaCa-2 cells after MGDG (25 μM for 24 h), radiation (8 Gy for 12 h), or both. **a** Detection of DNA damage by γ-H2AX foci staining. **b** Quantification of γ-H2AX-positive cells. **c** Comet assay for detection of DNA fragments. *P < 0.05

The effects of MGDG and radiation on the cellular DNA damage response was evaluated by quantifying γ-H2AX foci in MIAPaCa-2 cells. Compared to irradiation alone, combination treatment increased the number of γ-H2AX foci in MIAPaCa-2 cells (Fig. 5a, b). The results indicate that MGDG and irradiation induce DNA damage in a synergistic manner.

We performed the alkaline comet assay to detect defects in DNA repair. At 30min, the proportion of comet tails indicating DNA fragments was higher in the combination treatment (46.5%) than in the MGDG (3.2%) and irradiation (30.5%) groups (*P* < 0.05) (Fig. 5c). At later time points, most cells had completed DNA repair or had undergone apoptosis. These results indicate that MGDG potentiated the suppressive effects of radiation on DNA repair.

### Cell cycle distribution

**Fig. 6:**
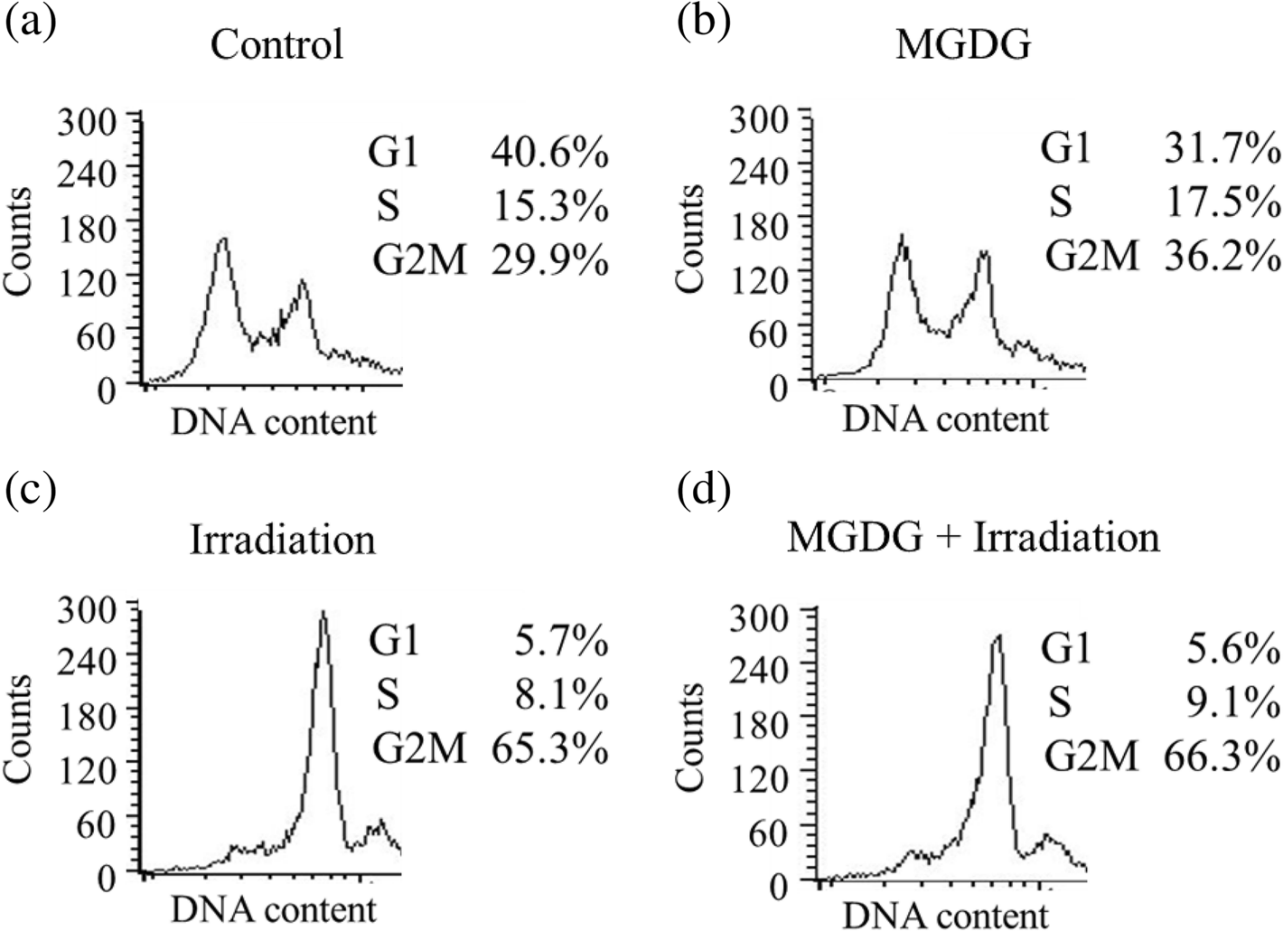
Cell cycle distribution in MIAPaCa-2 cells after treatment with MGDG, radiation or both. **a** Control, **b** MGDG (25 μM for 24 h), **c** radiation (8 Gy for 12 h), and **d** combination of 25 μM MGDG for 24 h and 8 Gy radiation for 12 h

To determine whether cell cycle was affected by MGDG or irradiation, MIAPaCa-2 cell cycle distribution was analyzed by flow cytometry. MGDG induced a slight increase in the G2/M fraction (29.9–36.2%) (Fig. 6a, b), while irradiation caused G2/M arrest (29.9–65.3%) (Fig. 6a, c). The cell cycle distribution following combination treatment was similar to that of irradiation alone (Fig. 6c, d). These results indicate that MGDG does not act synergistically with radiation to induce cell cycle arrest.

### Tumor growth inhibitory effect

**Fig. 7:**
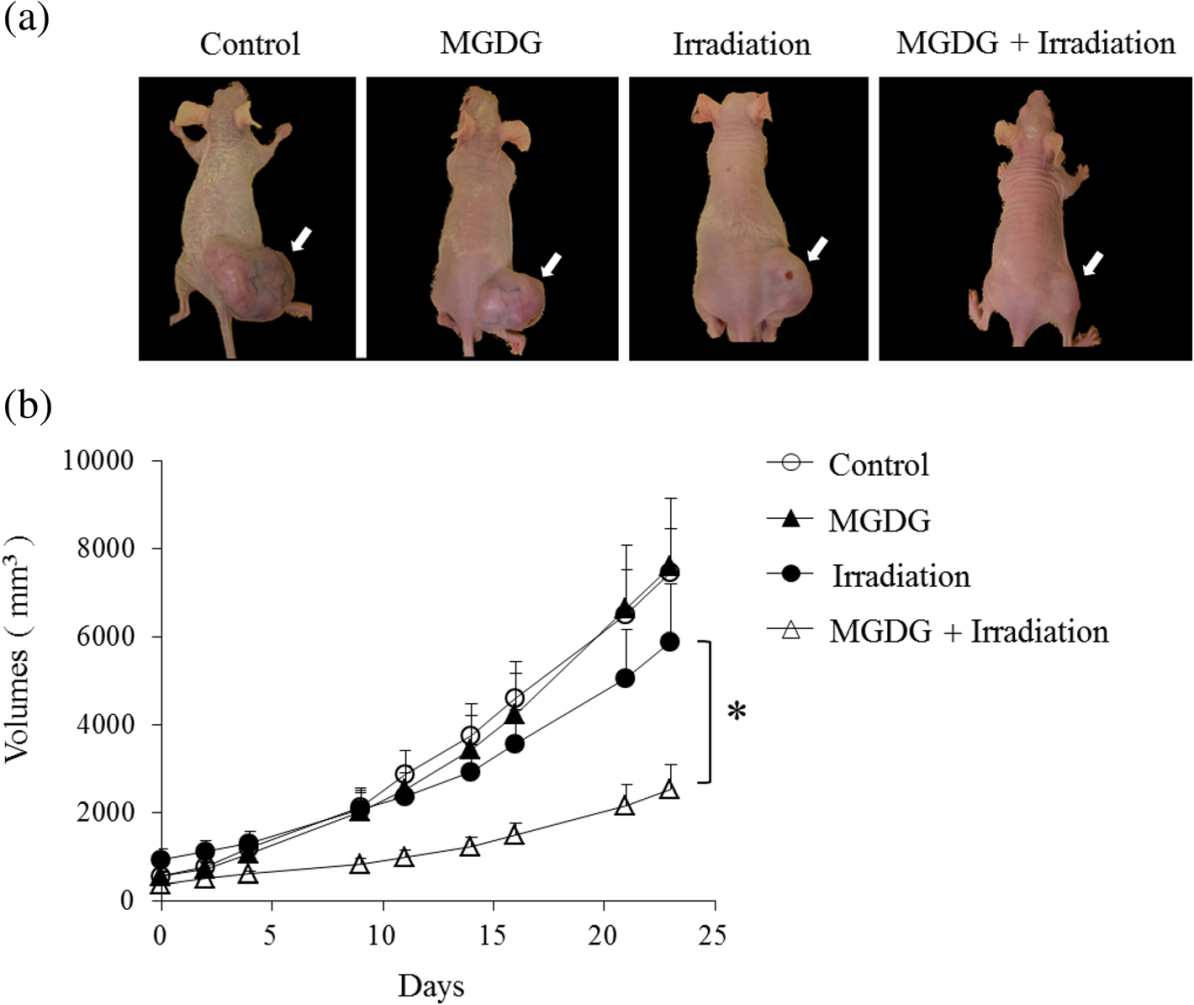
Tumor growth inhibitory effect of MGDG combined with irradiation. **a** Tumor appearance in xenografts 23 days after treatment (arrowhead). **b** Sequential measurements of tumor size after each treatment. Data are shown as mean ± SE. * *P* < 0.05

The effects of MGDG and radiation were assessed in a mouse xenograft model using MIAPaCa-2 cells. After 23 days, the tumor volumes were 7475.3 ± 986.1 mm^3^ (control), 7598.8 ± 1532.0 mm^3^ (MGDG), 5892.7 ± 1313.3 mm^3^ (radiation), and 2539.8 ± 552.7 mm^3^ (MGDG and radiation) (Fig. 7a, b). The tumor growth inhibitory effect was greater in mice receiving intratumoral injection of MGDG combined with irradiation as compared to either of these approaches alone (*P* < 0.05) (Fig. 7b).

## Discussion

In our previous study, MGDG was found to selectively inhibit mammalian pols α, γ, δ and ε while having no effect on other mammalian pols, such as those related to repair (β, η, ι, κ, λ and μ) or terminal deoxynucleotidyl transferase activities [21]. In the present study, we found that MGDG suppressed proliferation of various cancer cell types, including pancreatic cancer cells. Given that the 50% lethal dose (LD_50_) for suppression of cell growth by MGDG is nearly equivalent to the IC_50_ for inhibition of polymerase activities, we speculate that MGDG penetrates cancer cells and inhibits the activities of mammalian nuclear DNA repair pols δ and ε as well as the mitochondrial DNA repair pol γ, leading to radiosensitization and cell growth suppression. We also compared the growth-inhibitory effects of MGDG with those of other spinach glycoglycerolipids in several cancer cell lines (Table 1). Interestingly, the MGDG had more potent cytotoxic effects than these two compounds in all cell types examined, consistent with a previous report [9].

Pancreatic cancer is a lethal disease; the 5-year survival rate is approximately 3–7% following diagnosis [1, 2]. Surgical resection is the only curative treatment but the majority of patients are diagnosed at late, inoperable stages. Locally advanced pancreatic cancer (LAPC) is resistant to many forms of chemoradiotherapy (CRT) [3]. CRT with concurrent 5-fluorouracil was previously considered as a standard treatment for LAPC [25, 26]. More recently, based on favorable responses to GEM-based chemotherapy [27, 28] and the observation that GEM is a potent radiosensitizer [29], GEM-concurrent CRT has been used to treat LAPC [30, 31], with improved survival rates. Since conventionally fractionated chemoradiotherapy is associated with 1-year local control rates of only 40–60%, a strategy to enhance the effects of radiation is necessary. To this end, our observation of radiosensitization by MGDG suggests that it can be a possible treatment option for LAPC. A recent study reported successful results by combining GEM with proton therapy to treat LAPC; the 1-year local progression-free and overall survival rates were 81.7 and 76.8%, respectively [32]. Alternatively, stereotactic body radiation therapy can potentially achieve high local controllability in the management of pancreatic cancer [33, 34]. However, these methods have also been associated with a high incidence of acute morbidity [32, 33]. Given that MGDG enhances the effects of GEM in pancreatic cancer cell lines [21] as well as those of irradiation in this study, the therapeutic efficacy of MGDG combined with a lower dose of GEM or with radiotherapy should be tested as a means of reducing the morbidity of chemoradiotherapy for LAPC.

Most chemotherapeutic agents do not significantly improve the survival of pancreatic cancer patients [35]. Dysregulation of pro-apoptosis signaling is a survival mechanism for pancreatic cancers. Given that nearly every step of carcinogenesis inhibits apoptosis, the development of therapeutic strategies that target this process is essential for effective treatment of pancreatic cancer [36]. MGDG was recently introduced as a novel anticancer agent that can potentially increase apoptosis rates in human cancer cell lines. We previously showed that combining MGDG and GEM induced cell death and apoptosis [21]. In this study, MGDG showed dose-dependent cytotoxicity in pancreatic cancer cell lines, as evidenced by increased mitochondrial cytochrome c release and activation of caspase-3 followed by PARP, which resulted in apoptosis. Radiation causes DNA damage and kills mammalian cells by inducing single- and double-strand breaks in DNA [37]. The fact that the combination treatment showed greater cytotoxicity in vitro and in vivo than either approach alone indicates that the inhibitory effects of radiation on DNA are synergistically enhanced by MGDG.

MGDG alone or combined with radiation had negligible cytotoxicity in NHDFs as determined by the MTT assay, which is consistent with previous findings [21]. We used this as opposed to the colony forming assay since these cells did not form measurable colonies. However, one limitation of this study is that the possible side effects of MGDG were not fully investigated; specifically, the toxicity at high doses caused by its inhibition of DNA polymerase activity warrants further investigation. The side effects of MGDG are presumably similar to those caused by pyrimidine analogs such as GEM and cytarabine. GEM is widely used to treat various types of malignancy including non-small cell lung and breast cancers and is a key drug for the treatment of pancreatic cancer [38]. An in vivo model in which various doses are tested followed by a phase I clinical study can clarify the potential side effects of MGDG. Another limitation is that administration of MGDG was tested by intratumoral injection. In an earlier study, tumor growth was slightly inhibited by the oral administration of spinach glycolipid fractions containing MGDG, DGDG and SQDG [8]; however, oral administration of a single compound (e.g., MGDG) combined with irradiation has not been previously assessed. Using different modes of administration could potentially clarify the efficacy of MGDG for the treatment of pancreatic cancer.

## Conclusions

This is the first report providing evidence that MGDG enhances the cytotoxic effects of radiation in pancreatic cancer cells in vitro and in vivo. This effect is likely associated with stimulation of mitochondrial cytochrome c release. Combined treatment with MGDG and radiation synergistically suppressed tumor growth in vivo. Our findings indicate that this therapeutic combination can be an effective strategy for the treatment of pancreatic cancer.

## Abbreviations

Bcl-2: B cell lymphoma-2
CRT: Chemoradiotherapy
DGDG: Digalactosyl diacylglycerol
DMSO: Dimethylsulfoxide
FITC: Fluorescein isothiocyanate
GEM: Gemcitabine
IC_50_: Half maximal (50%) inhibitory concentration
LAPC: Locally advanced pancreatic cancer
MGDG: Monogalactosyl diacylglycerol
MTT: 3-(4,5-dimethylthiazol-2-yl)-2,5-diphenyltetrazolium bromide
NHDF: Primary normal human dermal fibroblast
PARP: Poly (ADP-ribose) polymerase
PBS: Phosphate-buffered saline
PBST: PBS containing 0.05% Tween
SQDG: Sulfoquinovosyl diacylglycerol
γ-H2AX: Phosphorylated histone 2AX
